# Students’ School and Psychological Adjustment in Classrooms with Positive and Negative Leaders

**DOI:** 10.1007/s10964-023-01937-w

**Published:** 2024-01-06

**Authors:** Zhe Dong, Gijs Huitsing, René Veenstra

**Affiliations:** Department of Sociology and Interuniversity Center for Social Science Theory and Methodology, University of Groningen, Groningen, the Netherlands

**Keywords:** Leadership, Victimization, Children, Adjustment

## Abstract

Positive and negative leadership styles may influence classroom norms and be related to the school and psychological adjustment of children in general, and victims in particular. This study tested the relation between leadership styles and children’s adjustment, and the moderating effects of leadership on the association between self-reported victimization and school and psychological adjustment (self-esteem, social anxiety, and depressive symptoms); and it tested for potential gender differences. Classrooms were classified into those with only positive leaders, only negative leaders, both positive and negative leaders, and without leaders. The sample contained 8748 children (*M*_age_ = 10.05, *SD* = 1.22; 51.2% girls) from 392 classrooms, in grades three to six, in 98 Dutch schools. Multilevel analysis revealed that, in general, children in negative leader classrooms experienced lower school well-being than children in other classrooms. In positive leader classrooms, male and female victims had lower school well-being. For psychological well-being, female victims had lower self-esteem and more depressive symptoms in positive leader classrooms. Male and female victims in negative leader classrooms did not suffer from additional maladjustment. These results demonstrate that negative leadership styles are related to lower school well-being of all children, whereas victimization in classrooms with positive leaders is negatively related to male and female victims’ school well-being and girls’ psychological adjustment (depressive symptoms); this is in line with the ‘healthy context paradox’.

## Introduction

Leadership styles in late childhood have been distinguished as positive and negative (Dong et al., 2023). Positive leaders show the highest involvement in defending and low involvement in bullying behaviors, whereas negative leaders show moderate involvement in defending and high involvement in bullying. Leadership styles play an important role in framing social norms and shaping the social environment of classmates (Inceoglu et al., 2018). However, it is unknown how leadership styles relate to the school and psychological adjustment of children in general, and victims in particular. For all classmates, leadership styles influence the classroom environment through viable yet distinct strategies (i.e., prosocial or aggressive) to exert influence (Maner & Case, 2016). For victims, specifically from an attributional perspective (Weiner, 1982), the styles in which leaders influence the classroom environment – through positive or negative patterns – may be related to their well-being by altering the explanation of why they are victimized. In addition, boys and girls with different attributional styles may respond differently to bullying (Shelley & Craig, 2010). Therefore, the purpose of this study was to examine how leadership styles are related to the school and psychological adjustment of classmates in general, and victims in particular, and explore gender differences in response to these leadership styles. To this end, children were classified as being in classrooms with only positive leaders, only negative leaders, both positive and negative leaders, and without leaders. The aim in doing this was to understand how popular leaders’ strategies are associated with the adjustment of children in general, and victims in particular. School adjustment is defined as children’s attitudes toward attending school and whether they feel safe and secure at school (Belfi et al., 2012). Psychological adjustment includes the presence of life satisfaction and the absence of psychological distress or symptoms (in terms of self-esteem, social anxiety, and depressive symptoms; Houben et al., 2015).

### Leadership and Classroom Climate

Leadership has been defined as a process that occurs between leaders and classmates (Northouse, 2018). The consequences of leadership can be elucidated through the variations of positive and negative styles, each associated with its own set of processes, motivations, and influence tactics (Kakkar & Sivanathan, 2021). Popular leaders set norms that lead to their social prominence and disproportionately affect others (Farmer, 2000); they are critical in fostering social significance in classrooms and are ideally positioned to help peers in achieving their social goals (Maner & Mead, 2010).

Positive leadership is perceived as being prosocial (Andrews, 2020), establishing and maintaining favorable relationships with others (Dong et al., 2023), and getting along with the group (Hu et al., 2019). Positive leaders typically help the group achieve its goals and use power to benefit others (Hawley, 2003). An example would be sharing information, knowledge, and skills that are valuable to the group (Maner & Case, 2016). Such influence is reciprocated with respect, deference, and positive relationships (Maner & Mead, 2010). Perpetrators may be more likely to reduce bullying behaviors for fear of disapproval from positive leaders. Therefore, classmates may enjoy a more nurturing atmosphere in classrooms with positive leadership than in classrooms without any leadership style.

Negative leadership, in contrast, involves bi-strategic (Dong et al., 2023) and coercive strategies (Hartl et al., 2020), with the aim of getting ahead when interacting with others (Hu et al., 2019). To control power and rank, negative leaders use cooperation and benevolence to hide the truth of their coercive accomplishments and intimidating methods (Maner & Case, 2016). As a result, they prioritize self-serving goals by pressuring others to follow them, especially when the group’s goals and interests conflict with their own goals and interests (Maner & Mead, 2010).

In classrooms with both positive and negative leadership styles, negative leaders are more likely to prioritize their own power and maintain social status, and battle with positive leaders to protect power levels and maintain the power gap in the classroom (Maner & Mead, 2010). Therefore, the influence tactics in negative and mixed leader classrooms induce fear based on a sense of psychological threat (Cheng et al., 2013). Thus, it is more difficult for children to enjoy positive peer relationships in classrooms with negative or mixed leadership styles, than in classrooms without leaders. Therefore, it was expected that children in positive leader classrooms would have better school and psychological well-being than those in negative leader or mixed leader classrooms (Hypothesis 1).

### Leadership Styles and Victimization

An important question is how victims, in particular, feel in classroom contexts characterized by positive, negative, or mixed leadership styles. Persistent victims in classrooms with clear anti-bullying norms feel paradoxically more hopeless and are more pessimistic about themselves and their environment; this has been described as the “healthy context paradox” (Garandeau & Salmivalli, 2019, Huitsing et al., 2019). Victims try to understand why they are victimized, and the dimensions of their subjective attribution processes are associated with their school and psychological well-being (Schacter & Juvonen, 2015). According to attribution theory (Weiner, 1982), attribution processes are related to the locus (whether the cause of victimization is internal or external to the victim), controllability (whether the cause of victimization can be changed), and stability (whether the cause of victimization is stable or changes over time) of victimization.

In a high bullying norm context, victimization is more common, and victims can attribute their situation to this context (Schacter & Juvonen, 2015). That is, they are more likely to conclude that they are unlucky to be in the same classroom as negative leaders, adopting an *external* perspective. Negative leaders can exhibit a dual nature, oscillating between defensive and bullying behaviors (*unstable*), making change an arduous task. The actions of such leaders prove difficult to manage, rendering them seemingly *uncontrollable*. Furthermore, attributing negative events to external, unstable, and specific causes (Peterson et al., 1993) holds promise. This perspective suggests that attributing negative events to external factors may reduce harm and protect self-esteem (Graham & Juvonen, 2002). Consequently, victims may hold out the hope that moving to a classroom free of negative leadership would bring an end to their victimization.

In contrast, in classrooms characterized by positive leadership styles in an environment of low tolerance for bullying, an unexpected pattern emerges in which victimization is paradoxically associated with increased maladjustment. The establishment of anti-bullying norms in these settings significantly influences how victims perceive the reasons for bullying (Graham & Juvonen, 1998). In classrooms with high anti-bullying norms, victims are more likely to attribute ongoing bullying to their own actions rather than to the classroom environment, adopting an *internal* perspective. Despite the presence of supportive leaders, they find themselves targeted by bullies, a situation perceived as *stable*. Even the involvement of high-status peers proves ineffective in changing their predicament, leaving these victims with a sense of powerlessness to effectively change the situation, given their isolation from peers who share similar experiences. This powerlessness may lead them to lose hope that the torment will ever end, and thus to view it as *uncontrollable*. In addition, these victims face the challenge of having fewer classmates who can relate to their experiences. As a result, they are more likely to blame themselves, further exacerbating their adjustment difficulties. Previous research has shown that adopting an internal attribution style is associated with the development of depressive symptoms (Shelley & Craig, 2010). Victims who attribute their experiences to internal, stable, and uncontrollable causes are more prone to maladjustment (Schacter & Juvonen, 2015). This maladjustment manifests itself in various ways, including low self-esteem (Laninga-Wijnen et al., 2021) and depressive symptoms (Yun & Juvonen, 2020).

In classrooms with both positive and negative leadership styles, negative leaders may be less likely to stop bullying behaviors because they do not fear the disapproval of positive leaders (Maner & Mead, 2010). Furthermore, bad may be stronger than good (Baumeister et al., 2001), and victims may pay more attention and be more sensitive to negative rather than positive primes (Lansu & Troop-Gordon, 2017). Thus, the aggressive behaviors associated with negative leadership may attract more attention than the prosocial behaviors associated with positive leadership. All of the above suggests that victims in such classrooms will be more likely to be maladjusted. Based on this reasoning, it was expected that *victims* in positive leader classrooms and mixed leader classrooms would have more school and psychological maladjustment than victims in negative leader classrooms or classrooms without leaders (Hypothesis 2).

### Differences between Boys and Girls

Gender differences have not received much attention in attribution research (Schacter & Juvonen, 2015). Girls generally have lower adjustment than boys in response to bullying (Rueger & Jenkins, 2014). After the bullying has stopped, the maladjustment has been found to persist more often for girls than for boys (Rueger et al., 2011). The attributional styles of girls, but not boys, may lead to depressive symptoms after victimization, because girls expect that bullying could be inevitable (Shelley & Craig, 2010). Previous studies applying attribution theory tested for mean-level gender differences in maladjustment (Huitsing et al., 2019; Pan et al., 2021), but it remains unclear whether victimized boys and girls in different types of classrooms respond differently to their victimization in terms of adjustment problems. To shed light on the gender differences in adjustment in response to victimization, this study examined the possible differences for boys and girls.

## Current Study

Because positive and negative leadership styles lead to different classroom norms, it is unclear how they are related to the well-being of children in general, and victims in particular. This study examined which types of classrooms foster a more favorable classroom climate. First, a typology of classrooms based on leadership types was developed. Using a person-centered approach based on the number of peer nominations received for leadership, popularity, defending, and bullying, this study was able to differentiate between individuals who exhibited positive and negative leadership qualities. These distinct types of leaders were used to categorize classrooms as having only positive leaders, only negative leaders, and both, leaving classrooms without leaders. A multilevel framework was then used to test whether *all children* in positive leader classrooms had better school and psychological adjustment than those in negative leader classrooms or mixed leader classrooms (Hypothesis 1). Next, the study tested whether the association between victimization and maladjustment differed across classroom types. In positive leader classrooms, it was expected that victims would attribute the bullying more often to themselves, leading to adverse effects. In classrooms with only negative leaders, victims were expected to attribute the bullying to negative leaders, and to be less vulnerable to the adverse effects. In classrooms with both positive and negative leaders, negative leaders were expected to be more dominant in prioritizing their power and maintaining social status, and victims were expected to experience adverse effects. Thus, it was tested whether *victims* would have worse adjustment in positive leader classrooms and mixed leader classrooms and less maladjustment in classrooms with only negative leaders (Hypothesis 2). Possible differences in the negative consequences of victimization for boys and girls were examined as well.

## Methods

### Procedure

Data used in this study stem from the KiVa anti-bullying program in the Netherlands, and were all collected in October 2012 (Huitsing et al., 2020; Veenstra et al., 2020). Before the data collection, an information guide and consent forms were sent to parents, with the request to allow their children to participate. Passive parental and student consent was obtained: parents could opt their children out of participation, and children themselves could also opt out of the assessment at any time. The response rate at each wave exceeded 95%. Children completed the questionnaires online in the school computer labs, during regular school hours, under teachers’ guidance, and they could ask teachers for help when necessary. Children who missed the scheduled day of data collection could participate another day within a month. The questions of each scale were presented in random order to avoid the possibility that the order might systematically affect the results.

For the peer nomination questions, a list of the names of classmates was provided for the children to choose from: they were asked to nominate an unlimited number of classmates for all peer nomination questions. Children could nominate the same peer for more than one question, and were allowed to nominate absent peers. To take differences in classroom size into account, the number of peer nominations each child received from participating classmates was converted into proportion scores.

### Sample

The final sample consisted of 8748 children (*M*_age_ = 10.05, *SD* = 1.22; 51.2% girls) from 392 classrooms (mean classroom size was 23.50, *SD* = 6.11) in 98 Dutch schools, in grades three to six (Dutch grades five to eight). The ethnic background of the students was reported to be 80.1% Dutch, 2.9% Moroccan, 1.8% Turkish, 2.6% Surinamese, and 1.1% Dutch Antillean. The remaining 11.6% of the children reported some other Western (6.1%) or non-Western (5.5%) ethnicity.

### Measures

#### Peer-reported leadership, popularity, bullying, and defending

To ensure that the children understood the meaning of leadership, the concept was explained (“Do you know what a good leader is? A leader is someone who often determines what needs to be done. Such as the captain of a team or a coach. They often say what others have to do”). The question, “Are there children in your class who are leaders? Which classmates are good leaders?” was used to indicate leadership. To ensure that the children understood the meaning of popularity, this concept was explained (“Popular children are children that others want to hang out with. Popular children are cool”). The question, “Which classmates are popular?” was used to indicate popularity. To measure bullying and defending, children were first asked whether they were being victimized on any of the 11 self-reported Olweus’ (1996) bully/victim items (concerning several forms of victimization). After watching an instructional video, in which the definition of bullying was explained (i.e., not the same as a fight between two people who are equally strong; repeated harassment of another child, with the victim having problems defending him or herself), participants responded to the bully/victim questionnaire. If they indicated that they had been victimized at least once on any item, they were asked whether they were victimized by classmates, other students from the school, or others outside the school. If children reported being victimized by classmates, they were asked, “Who starts when you are victimized?” in order to indicate their bullies. Defending was explained (“Defending is helping, supporting, or comforting victimized students”), and victimized children were asked, “Which classmates defend you when you are victimized?” in order to indicate their defenders. Proportion scores for the numbers of nominations children received from their classmates were calculated for leadership, popularity, bullying, and defending.

#### Self-reported victimization

Self-reported victimization was measured using the Revised Olweus Bully/Victim Questionnaire (Olweus, 1996). Participants responded to a global question (“How often have you been bullied during the past couple of months?”) and seven specific items related to physical, verbal (two items), relational (two items), material (i.e., taking or breaking others’ property), and cyberbullying. This is a five-point scale (0 = *not at all*, 1 = *once or twice*, 2 = *two or three times a month*, 3 = *about once a week*, 4 = *several times per week*), with Cronbach’s alpha = 0.87.

#### Well-being at school

Well-being at school was measured using a seven-item scale (Huitsing et al., 2019; Kärnä et al., 2011), including general liking of school (e.g., “I like it at school”) and feelings of safety (e.g., “I feel safe at school”). This is a four-point Likert-type scale (0 = never, 3 = always), with Cronbach’s alpha = 0.83.

#### Self-esteem

Self-esteem was measured using a five-item scale derived from the Rosenberg Self-Esteem Scale (Rosenberg, 1965), with only positively worded items applied to questions for this age group (e.g., I feel that I am a person of worth, at least on an equal plane with others). This is a five-point Likert-type scale **(**0 = never, 4 = always), with Cronbach’s alpha = 0.84.

#### Social anxiety

Social anxiety was measured using a scale derived from the Social Phobia Screening Questionnaire (Furmark et al., 1999). A sample item is, “I am scared to be together with others during the break”. The seven items were answered on a five-point Likert-type scale (0 = never, 4 = always), with Cronbach’s alpha = 0.72.

#### Depressive symptoms

Depressive symptoms were measured using a scale derived from the Major Depressive Disorder Scale (Chorpita et al., 2000). A sample item is, “I feel nothing is much fun anymore.” The nine items were answered on a four-point Likert-type scale (0 = never, 3 = always), with Cronbach’s alpha = 0.81.

#### Control variables

Three ordered dummies for grades were used as control variables: being in grade four or higher (children in grade three = 0, children in grade four or higher = 1), being in grade five or higher (children in grades three and four = 0, children in grades five and six = 1), and being in grade six (children in grade five or lower = 0, children in grade six = 1).

### Analytical Strategy

**Fig. 1:**
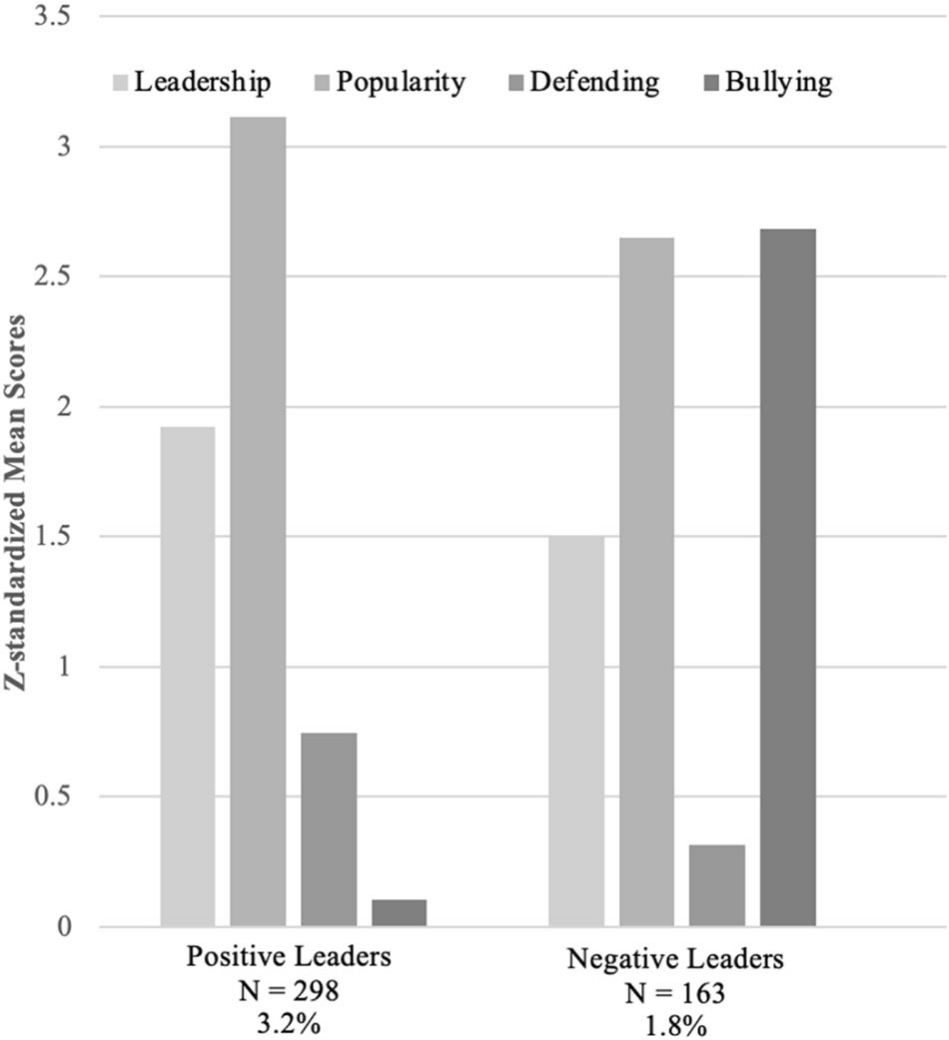
Profiles of positive and negative leaders (z-standardized mean scores)

To identify positive and negative leaders, latent profile analysis was applied based on the peer nomination scores for leadership, perceived popularity, and prosocial (defending) and antisocial (bullying) behaviors (Dong et al., 2023). The seven-profile solution had the best fit statistics; these were labeled according to the estimated z-standardized mean indicator variables: (1) positive leaders (3.2%, *N* = 298, 58.1% boys), (2) negative leaders (1.8%, *N* = 163, 82.5% boys), (3) defenders (8.9%, *N* = 816, 38.7% boys), (4) popular children (8.8%, *N* = 811, 57.6% boys), (5) bullies (7.9%, *N* = 727, 73.2% boys), (6) extreme bullies (1.9%, *N* = 176, 84.1% boys), and (7) modal children (67.5%, *N* = 6222, 45.0% boys). Positive and negative leaders were both high in leadership and popularity, but they differed in defending and bullying. *Positive leaders* were characterized by the highest levels of defending and the lowest levels of bullying, and *negative leaders* showed moderate levels of defending and high levels of bullying (see Fig. 1 for more details). To examine the relation between positive and negative leadership styles and the adjustment of the leaders’ classmates, only the children included in the remaining five profiles (95.0%, *N* = 8748, 48.8% boys) were used as the sample for this study. Four modal children were removed from the dataset because they had missing data on all variables.

The classrooms were then divided into four categories based on their leadership styles. The classrooms had only positive leaders (positive leader classrooms, 27.8% of classrooms, with 2361 children in total, 48.1% boys), only negative leaders (negative leader classrooms, 15.8% of classrooms, with 1384 children in total, 48.3% boys), both positive and negative leaders (mixed leader classrooms, 12.0% of classrooms, with 970 children in total, 48.7% boys), or no leaders (leaderless classrooms, 44.4% of classrooms, with 4035 children in total, 49.3% boys). This information was used to construct three dummies for being in positive leader classrooms, negative leader classrooms, and mixed leader classrooms, leaving leaderless classrooms as the comparison category.

Analyses were conducted in two steps. First, the descriptive information for adjustment outcomes was compared between types of classrooms using univariate ANOVA, including post hoc Scheffé tests. Second, multilevel regression models were estimated to determine whether there were significant differences in children’s adjustment in classrooms with different leadership styles. Interactions with gender were added to examine whether the effects of positive and negative leadership styles on the school and psychological well-being of children in general, and victims in particular, differed by gender. The two models were tested at two levels, with students (level 1) nested within classrooms (level 2) using Mplus 8.3 (Muthén & Muthén, 2012). The analyses were divided into three steps for each model. First, an empty model was used to obtain intra-class correlations (ICC, the proportion of total variance between classrooms) of the outcome variables of school well-being, self-esteem, social anxiety, and depressive symptoms; this served as the reference model. Next, ordered dummies for grades, the self-reported victimization scale, and the interaction between gender and self-reported victimization were included as Level 1 indicators in Model 1; and positive leader classrooms, negative leader classrooms, and both leader classrooms, along with their interactions with gender, were included as Level 2 indicators in Model 1. Leaderless classrooms were used as the comparison group. Model 1 was used to examine whether children in positive leader classrooms had better school and psychological adjustment than children in negative leader classrooms and classrooms with both positive and negative leaders (Hypothesis 1). In Model 2, the interaction effects of “Victimization × Classroom Subtypes”, and “Victimization × Classroom Subtypes × Gender” were included to test the extent to which the effects of victimization on school and psychological adjustment differed across classroom types and gender. These interaction models tested whether victims, in particular, would have worse adjustment in positive leader classrooms and mixed leader classrooms and less maladjustment in negative leader classrooms (Hypothesis 2).

There were no missing data for the classroom subtypes and peer nomination variables, because all children received nominations from their classmates. Missing data were low for self-reported victimization (*N* = 52, 0.6%), school well-being (*N* = 51, 0.6%), self-esteem (*N* = 60, 0.7%), social anxiety (*N* = 110, 1.3%), and depressive symptoms (*N* = 111, 1.3%). Full information maximum likelihood (FIML) with maximum MLR estimation was used for missing data in M*plus*.

## Results

### Descriptive Statistics and Correlations

Table 1 shows that children had moderate levels of school well-being (*M* = 2.09, *SD* = 0.56) and self-esteem (*M* = 2.91, *SD* = 0.88), and low levels of social anxiety (*M* = 0.88, *SD* = 0.74) and depressive symptoms (*M* = 0.67, *SD* = 0.51). Self-reported victimization was significantly related to increased levels of all variables measuring school and psychological maladjustment, for both boys and girls.

**Table 1:** Descriptive statistics and correlations for study variables for girls (above the diagonal) and Boys (below the diagonal)

Variables	*M*	*SD*	1	2	3	4	5
1. Self-reported victimization	0.48	0.69	1	−0.32	−0.18	0.22	0.36
2. School well-being	2.09	0.56	−0.25	1	0.40	−0.22	−0.36
3. Self-esteem	2.91	0.88	−0.19	0.35	1	−0.21	−0.33
4. Social anxiety	0.88	0.74	0.24	−0.15	−0.17	1	0.35
5. Depressive symptoms	0.67	0.51	0.38	−0.30	−0.24	0.34	1

All correlations are significant at <0.001

### Univariate Analysis

Table 2 presents descriptive information and post hoc Scheffé tests for the four types of classrooms. In the 109 classrooms with positive leaders, there were a total of 208 positive leaders. The 62 classrooms with negative leaders had a total of 94 negative leaders. In addition, 47 classrooms had both positive and negative leaders, with 90 positive leaders and 69 negative leaders. Positive leader classrooms (71.9% in grades five or six) and mixed leader classrooms (86.6% in grades five or six) were more common in the higher grades, whereas negative leader classrooms (57.2% in grades three or four) and leaderless classrooms (71.1% in grades three or four) were more common in the lower grades. Children in positive leader classrooms had lower self-reported victimization, higher school well-being, and lower depressive symptoms than children in negative leader classrooms.

**Table 2:** Study variables per cluster: means (or percentages) and ANOVAs

Type	Positive leader classrooms	Negative leader classrooms	Mixed leader Classrooms	Leaderless classrooms	Cluster Differences	With Control for Sex
1. Number of classrooms	109	62	47	174		
2. Number of Positive Leaders (M ± SD)	208 (1.91 ± 1.40)	0	90 (1.91 ± 1.02)	0		
3. Number of Negative Leaders (M ± SD)	0	94 (1.52 ± 0.84)	69 (1.47 ± 0.80)	0		
4. Number of children	2361 (48.1% boys)	1384 (48.3% boys)	968 (48.7% boys)	4035 (49.3% boys)		
5. Children in grade 3	11.6%	21.9%	1.8%	39.2%		
6. Children in grade 4	16.5%	35.3%	11.6%	31.9%		
7. Children in grade 5	32.0%	28.9%	39.8%	17.5%		
8. Children in grade 6	39.9%	13.9%	46.8%	11.4%	χ^2^ = 1998.99^**^	
9. Mage	10.58^c^	9.92^b^	10.91^d^	9.58^a^	*F* = 606.23^**^	*F* = 454.96^**^
10. Self-reported victimization	0.40^a^	0.59^b^	0.46^a^	0.54^b^	*F* = 26.44^**^	*F* = 21.42^**^
11. School well-being	2.13^b^	2.00^a^	2.02^a^	2.11^b^	*F* = 22.99^**^	*F* = 25.55^**^
12. Self-esteem	2.95^a^	2.89^a^	2.91^a^	2.90^a^	*F* = 1.90	*F* = 17.12^**^
13. Social anxiety	0.85^a^	0.91^a^	0.91^a^	0.87^a^	*F* = 2.57	*F* = 72.08^**^
14. Depressive symptoms	0.64^ab^	0.71^c^	0.69^bc^	0.63^a^	*F* = 9.27^**^	*F* = 8.05^**^

Variables 1-8 were not included in the ANOVA. Variables 9-14 were estimated from ANOVA Means in the same row that do not share superscripts differ at *p* < .05 in the Scheffé test

### Multilevel Models

Intra-class correlations were calculated in the empty models for school well-being, self-esteem, social anxiety, and depressive symptoms; these were 5.9, 1.9, 2.5, and 0.9%, respectively.

### The Main Effects Models

Table 3 presents the main effects of individual- and classroom-level predictors, using the leaderless classrooms as the comparison group. Table 3 presents all main effects for girls. Estimates for boys are discussed in the text only when they differ significantly from those for girls. Self-reported victimization was associated with lower school well-being (*b*_*girls*_ = −0.464, *p* < 0.001, 95% CI = [−0.506, −0.422]; *b*_*boys*_ = −0.341, *p* < 0.001, 95% CI = [−0.380, −0.302]), lower self-esteem (*b* = −0.232, *p* < 0.001, 95% CI = [−0.275, −0.189]), more social anxiety (*b* = 0.302 *p* < 0.001, 95% CI = [0.247, 0.357]), and more depressive symptoms (*b* = 0.486, *p* < 0.001, 95% CI = [0.445, 0.527]).

**Table 3:** Main models and cross-level interactions predicting school well-being, self-esteem, social anxiety, and depressive symptoms

	School well-being		Self-esteem		Social anxiety		Depressive symptoms	
	Model 1	Model 2	Model 1	Model 2	Model 1	Model 2	Model 1	Model 2
Intercept	0.416 (0.036)	0.398 (0.038)	−0.059 (0.033)	−0.099 (0.035)	0.066 (0.034)	0.088 (0.036)	−0.113 (0.030)	−0.098 (0.033)
*Individual level*								
Grade 4/5/6	−0.089 (0.038)*	−0.085 (0.038)*	0.127 (0.034)**	0.136 (0.035)**	−0.025 (0.038)	−0.026 (0.034)	−0.055 (0.012)	−0.056 (0.031)
Grade 5/6	−0.055 (0.038)	−0.053 (0.038)	0.013 (0.034)	0.023 (0.035)	0.006 (0.034)	0.008 (0.035)	−0.119 (0.031)**	−0.115 (0.031)**
Grade 6	−0.028 (0.035)	−0.038 (0.035)	−0.004 (0.033)	−0.006 (0.033)	−0.056 (0.032)+	−0.056 (0.033)	−0.045 (0.031)	−0.044 (0.031)
Victimization	−0.464 (0.021)**	−0.450 (0.033)**	−0.232 (0.022)**	−0.174 (0.033)**	0.302 (0.028)**	0.232 (0.022)**	0.486 (0.021)**	0.466 (0.032)**
Gender (Boy =1)	−0.173 (0.025)**	−0.176 (0.037)**	0.165 (0.036)**	0.183 (0.039)**	−0.367 (0.035)**	−0.381 (0.038)**	−0.066 (0.034)	−0.075 (0.036)
Victimization × Boy	0.123 (0.028)**	0.130 (0.046)**	−0.007 (0.030)	−0.039 (0.047)	−0.002 (0.042)	−0.007 (0.030)	0.004 (0.028)	0.011 (0.047)
*Classroom level* (Type of classroom)								
Positive leaders	0.019 (0.046)	0.083 (0.052)	−0.054 (0.041)	−0.001 (0.048)	0.022 (0.043)	−0.001 (0.049)	0.085 (0.037)*	0.027 (0.045)
Negative leaders	−0.177 (0.052)**	−0.189 (0.061)**	−0.030 (0.048)	0.031 (0.056)	0.006 (0.050)	−0.028 (0.057)	0.005 (0.043)	−0.006 (0.054)
Mixed	−0.110 (0.062)	−0.040 (0.072)	−0.041 (0.057)	0.040 (0.067)	0.006 (0.059)	−0.058 (0.068)	0.003 (0.051)	−0.006 (0.063)
*Cross-level interactions*								
Positive × Boy	0.006 (0.052)	0.002 (0.060)	0.078 (0.054)	0.035 (0.062)	−0.024 (0.052)	0.034 (0.061)	−0.066 (0.051)	0.006 (0.059)
Negative × Boy	0.024 (0.062)	0.045 (0.074)	0.019 (0.065)	0.011 (0.077)	0.029 (0.062)	−0.008 (0.075)	0.088 (0.061)	0.083 (0.072)
Mixed× Boy	−0.024 (0.071)	−0.013 (0.083)	−0.042 (0.074)	−0.056 (0.087)	0.015 (0.071)	0.059 (0.085)	0.045 (0.069)	0.025 (0.082)
Vict × PosCL		−0.156 (0.063)*		−0.143 (0.064)*		0.065 (0.064)		0.149 (0.062)*
Vict × NegCL		0.008 (0.062)		−0.119 (0.062)		0.076 (0.063)		0.016 (0.061)
Vict × MixedCL		−0.157 (0.082)		−0.182 (0.083)*		0.159 (0.083)		0.005 (0.081)
Vict × PosCL × Boy		0.022 (0.085)		0.103 (0.086)		−0.114 (0.088)		−0.175 (0.086)*
Vict × NegCL × Boy		−0.050 (0.087)		0.005 (0.088)		0.026 (0.090)		0.028 (0.089)
Vict × MixedCL × Boy		−0.033 (0.113)		0.025 (0.116)		−0.100 (0.117)		0.061 (0.115)
*Residual Variances*								
Within	0.854 (0.013)	0.820 (0.013)	0.940 (0.023)	0.915 (0.015)	0.902 (0.014)	0.868 (0.014)	0.853 (0.014)	0.815 (0.013)
Between	0.046 (0.009)	0.053 (0.012)	0.019 (0.007)	0.017 (0.008)	0.031 (0.008)	0.028 (0.010)	0.008 (0.006)	0.018 (0.008)
*Explained Variances %*								
Within	10.7	13.4	4.8	7.4	7.9	11.5	14.8	17.6
Between	25.6	20.7	15.2	19.5	7.1	13.1	43.9	33.5

*p < 0.05; **p < 0.01

At the classroom level, children in negative leader classrooms had lower school well-being than children in positive leader classrooms (*b* = −0.196, SE = 0.057, *p* = 0.001) or in leaderless classrooms (*b* = −0.177 SE = 0.052, *p* = 0.001). Children in mixed leader classrooms had somewhat lower school well-being than children in positive leader classrooms (*b* = −0.129, SE = 0.063, *p* = 0.039). Among girls, there was a higher prevalence of depressive symptoms in classrooms with positive leaders (*b* = 0.085, SE = 0.037, *p* = 0.022), compared with girls in leaderless classrooms. Boys in negative leader classrooms reported more depressive symptoms than boys in leaderless classrooms (*b* = 0.093, SE = 0.047, *p* = 0.047). No differences between classroom types were observed for self-esteem and social anxiety.

Consistent with the first hypothesis, both boys and girls in positive leader classrooms had higher levels of school well-being than those in negative or mixed leader classrooms. However, no differences in self-esteem or social anxiety were observed between boys and girls in positive leader classrooms and those in negative or mixed leader classrooms. Notably, girls in positive leader classrooms and boys in negative leader classrooms reported higher levels of depressive symptoms than their counterparts in other classroom types. This finding for girls contradicts the first hypothesis.

### Cross-Level Interaction Models

Model 2 in Table 3 shows interactions by regressing the adjustment variables on individual-level victimization in different classroom types. These models served to test the second hypothesis and explore gender differences. For girls, the negative effect of victimization on school well-being (*b* = −0.450, *p* < 0.001) was stronger in positive leader classrooms (*b* = −0.606, difference = −0.156, *p* = 0.013), but not in negative leader classrooms (*b* = −0.442, difference = 0.008, *p* = 0.898) or mixed leader classrooms (*b* = −0.607, difference = −0.157, *p* = 0.054). For boys, the negative effect of victimization on school well-being (*b* = −0.313, *p* < 0.001) was stronger in positive leader classrooms (*b* = −0.433, difference = −0.120, *p* = 0.040) and mixed leader classrooms (*b* = −0.493, difference = −0.180, *p* = 0.025), but not in negative leader classrooms (*b* = −0.340, difference = −0.027, *p* = 0.670).

Regarding self-esteem, the negative effect of victimization for girls (*b* = −0.174, *p* < 0.001) was stronger in positive leader classrooms *b* = (−0.317, difference = −0.143, *p* = 0.026) and in mixed leader classrooms (*b* = −0.356, difference = −0.182, *p* = 0.028), but not for girls in negative leader classrooms (*b* = −0.293, difference = −0.119, *p* = 0.056). Conversely, for boys, the negative effect of victimization (*b* = −0.206, *p* < 0.001) was not further differentiated by classroom type.

For social anxiety, the effects of victimization were not differentiated by classroom type for either girls (*b* =0.232, *p* < 0.001) or boys (*b* =0.290, *p* < 0.001).

The effect of victimization on depressive symptoms for girls (*b* =0.466, *p* < 0.001) was stronger in positive leader classrooms (*b* =0.615, difference = 0.149, *p* = 0.016), but not in negative leader classrooms (*b* =0.482, difference = 0.016, *p* = 0.800) or mixed leader classrooms (*b* =0.471, difference = 0.005, *p* = 0.951). The effect of victimization on depressive symptoms in positive leader classrooms was weaker for boys than for girls (difference *b* = −0.175, SE = 0.086, *p* = 0.042).

Consistent with the second hypothesis, both boys and girls who were victimized in positive leader classrooms experienced more school maladjustment. Female victims also experienced had more psychological maladjustment as a result of the healthy context paradox, as evidenced by lower self-esteem and increased depressive symptoms. The effects of victimization on depressive symptoms in positive leader classrooms differed significantly for boys and girls. Contrary to the second hypothesis, male victims did not experience difficulties in their psychological adjustment due to the healthy context paradox.

## Discussion

Previous research has neglected the potential impact of leadership styles, beyond fostering a positive classroom climate, on victims’ school and psychological adjustment through influence on their attributional processes. Multilevel analyses conducted on a large sample of 8748 children in 392 classrooms revealed that being in a classroom led exclusively by negative leaders was negatively associated with the overall school well-being of all children. In addition, it was found to correlate with increased depressive symptoms among boys. Being in classrooms with only positive leaders was not associated with all children’s school or psychological adjustment: consistent with attribution theory (Weiner, 1982), being victimized in classrooms with only positive leaders was more negatively related to school well-being for both victimized boys and girls, and further harmed girls’ self-esteem and depressive symptoms. These results are in line with the healthy context paradox effect, suggesting that victims have more maladjustment problems in classrooms with positive social norms.

Consistent with Hypothesis 1, all children in classrooms with only negative leaders had lower school adjustment than children in classrooms with only positive leaders or classrooms without leaders. This suggests that the negative leadership style is negatively related to the classroom context, because negative leaders rely on a combination of tactics to gain or maintain social influence, without concern for the experiences of classmates. A previous study found that leaders who use dominance tactics are more likely to think that their own success can only be achieved at the expense of others (Kakkar & Sivanathan, 2021). As a result, children in such classrooms have poorer peer relationships, are more likely to be victims of bullying, lack a sense of support, and have reduced feelings of readiness to enjoy school life (Laninga-Wijnen et al., 2021). Negative leaders were found to be associated with the overall classroom environment, which simply made school less enjoyable for all classmates. Being in classrooms led exclusively by negative leaders also showed a further association with depressive symptoms in boys. Surprisingly, girls in negative leadership classrooms did not show differences in psychological adjustment compared with girls in other classroom settings. A plausible explanation for this gender difference is that negative leaders are more likely to set norms for their same-gender peers, and because boys are more likely than girls to be negative leaders, their influence may have a more pronounced effect on male classmates than on female classmates. This finding underscores the importance of including gender interactions in the models.

The findings regarding children in classrooms with both types of leaders provide partial support for Hypothesis 1. In classrooms with both positive and negative leaders, both boys and girls exhibited lower school adjustment compared with boys in classrooms with only positive leaders. Contrary to Hypothesis 1, the presence of both types of leaders showed no additional association with children’s self-esteem, social anxiety, or depressive symptoms. A plausible explanation for this could be that the presence of positive leaders does not deter negative leaders from continuing their bullying behavior. While this has a negative impact on the academic well-being of other classmates, there was no additional impact on psychological adjustment.

### Leadership Styles and the Plight of Victims

The findings generally supported the second hypothesis, stating that victims would have more maladjustment problems in classrooms with positive leaders. First, both male and female victims in classrooms with positive leaders had lower school well-being than victims in classrooms without leaders or classrooms with only negative leaders. This finding is in line with the healthy context paradox, and highlights an unintended consequence of positive leadership. Being in a positive classroom context exacerbates the negative association between victims’ victimization and school well-being, revealing a moderate pathway through altering victims’ attributions of why they are bullied. It may be that victims in positive leader classrooms did not have the opportunity to share emotional problems with classmates who had undergone comparable life events (Kiuru et al., 2020). Thus, these victims were more likely to blame themselves, which in turn led to negative perceptions and a lower sense of school belonging.

Second, victims in classrooms with only negative leaders had comparable levels of school and psychological adjustment to victims in classrooms without leaders. It may be that they attribute their plight to the stable and uncontrollable contextual factors of the classroom, and that these victims attribute the reasons for their victimization to the negative classroom context as facilitated by the negative leaders (Schacter & Juvonen, 2015). Considering the findings for victims in positive leader classrooms, this may be evidence that victimization attributions lead to different adjustment pathways (Schacter & Juvonen, 2015).

Although it was hypothesized that being in the same classroom with both positive and negative leaders would have a detrimental effect on victims’ adjustment (Kakkar & Sivanathan, 2021), the results showed that the level of school adjustment was comparable to that of children in classrooms without leaders. Victims in mixed leader classrooms had better adjustment than expected, with the exception of victimized boys who experienced lower school well-being, and victimized girls, who experienced lower self-esteem. Previous experimental research has found that leaders tend to exclude group members who have the potential to influence their own leadership status, even if the exclusion threatens the group’s goals (Maner & Mead, 2010). It may be that the presence of positive and negative leadership styles increases intragroup competition. Further research could investigate the dynamic processes in classrooms with both positive and negative leaders, and could test whether victims are specifically targeted by the negative leaders or defended by the positive leaders.

### Gender Differences in Victims’ Attributional Styles

The analysis of the interaction between victimization, classroom type, and gender revealed that victimized girls in positive leader classrooms had significantly higher levels of depressive symptoms than boys in positive leader classrooms. This is consistent with earlier research, which found that the association between victimization and depressive attributions was stronger for girls than boys (Shelley & Craig, 2010).

For boys, victimization in positive leadership classrooms only exacerbated their school well-being; there were no further associations with their psychological adjustment. In contrast, for girls, experiencing victimization in positive leader classrooms led to lower self-esteem and more depressive symptoms, in addition to lower school well-being, compared with victimized girls in classrooms with no leaders. Boys who were victimized in mixed leadership classrooms had lower levels of school well-being; victimized girls in mixed leadership classrooms had lower levels of self-esteem. These findings suggest that victimization experiences trigger different attributional processes for boys and girls. Previous studies also found that victims had lower self-esteem when the number of victims in the classroom context was relatively limited (Garandeau et al., 2018; Huitsing et al., 2019), but they did not test for further gender differences. It may be that girls are more vulnerable to stressful peer experiences because of their stronger focus on interpersonal relationships (Rose, 2021). Girls with interpersonal stress are more likely to engage in (co-)rumination, which exacerbates the consequences of victimization for their psychological adjustment.

### Developmental Implications for Child Leadership

This study showed that children’s school and psychological adjustment is shaped by peer leaders during their formative years. Early identification and correction of inappropriate behavior by negative peer leaders has the potential to alleviate school and psychological adjustment problems. This stance is consistent with the concept of a positive snowball feedback loop, suggesting that targeted training during the sensitive developmental period will yield significant benefits (Day, 2011). Furthermore, this study highlighted the prevalence of males in both positive and negative leadership profiles, while girls appear to be more susceptible to the influence of leadership styles, potentially reinforcing gender role stereotypes. Finally, this study examined leadership development across age groups, and found an increase in positive leadership with age.

### Strengths, Limitations, and Directions for Future Research

This study has several strengths, including capturing the subtle moderating effects of positive or negative leadership styles on classmates’ well-being in an exceptionally large number of classrooms, and exploring gender differences in the context of the healthy context paradox for victims in particular. Most classrooms had a positive leader (27%), a negative leader (16%), or both (11%), and the study showed that leadership styles had relevant consequences for all children’s school adjustment, and for female victims’ psychological adjustment in particular. Future studies aiming to extend or replicate these findings need a large sample, because essentially the classroom level is the important level of analysis.

Notwithstanding the contributions of this study, the results must be interpreted with the following limitations in mind. A notable limitation of this study is the lack of measurement of attribution styles. Thus, it missed an opportunity to examine directly the cognitive processes underlying children’s responses to different leadership styles. Theoretically, attributional styles play a central role in shaping how victimization experiences are perceived and interpreted, which in turn can be associated with victims’ school and psychological adjustment. The lack of an assessment of attributional styles prevents a thorough understanding of why certain leadership styles might lead to distinct outcomes for different individuals and across gender. Similarly, classroom norms promoted by positive or negative leaders were only implicitly considered in our research. Future research that directly incorporates the assessment of attributional styles and classroom norms could provide valuable insights into the underlying mechanisms by which leadership styles moderate the association between victimization and well-being.

Second, the variable-oriented approach did not allow for the testing of children who experienced maladjustment as a result of being bullied by negative leaders specifically, or by other bullies or bully-victims. Further research could use a social network approach to examine the consequences of being bullied by negative leaders versus bullies or bully-victims, and the consequences of being defended by positive leaders, general defenders, or even negative leaders. The gender of the leader could also be included in a network approach. This relational approach could help in understanding the complexity of the link between positive and negative leadership and victims’ school and psychological adjustment.

Future research could also move beyond simply examining the relation between leadership styles and classmates’ adjustment, and focus on leaders’ own adjustment as well as the processes that link leadership adjustment to classmates’ adjustment. For example, research on adult leadership has focused on how leaders’ well-being is an important influence on followers’ well-being and vice versa (Inceoglu et al., 2018). Further research in schools could focus on coaching negative leaders to become positive leaders, as all children experience lower school well-being in classrooms with only negative leaders. In addition, future research could also explore the different experiences of victimization for boys and girls within classrooms characterized by relatively low levels of victimization. To our knowledge, this is the first study that examined the moderating effect of gender in relation to the healthy context paradox, and it suggests that victimized girls suffer more psychological maladjustment than victimized boys in an anti-bullying context.

## Conclusion

Positive and negative leadership styles are related to the school and psychological adjustment of children in general, and victims in particular. This study contributes to the literature by categorizing classrooms into positive leader classrooms, negative leader classrooms, both positive and negative leader classrooms, and classrooms without leaders. Children in classrooms with negative leaders had lower levels of school adjustment, and boys in classrooms with negative leaders had higher levels of depressive symptoms. Although there was no main effect of being in a classroom with positive leaders, the interactions showed that both male and female victims had lower school adjustment in positive leader classrooms. Furthermore, the consequences of victimization for depressive symptoms in classrooms with only positive leaders were stronger for girls than for boys, suggesting that the healthy context paradox is more prevalent for girls. Therefore, a practical recommendation is that future anti-bullying interventions should take into account the classroom leadership context of children in general, and victims in particular, and apply multiple solutions to facilitate changes in how bullying is addressed in different types of classrooms. Teachers should be more attuned to the leaders of their classrooms, and should verify whether their leadership style is focused on getting along with the group or getting ahead.
